# Molecular identification of forensically relevant Diptera inferred from short mitochondrial genetic marker

**DOI:** 10.3402/ljm.v8i0.20954

**Published:** 2013-05-17

**Authors:** Sanaa Mohamed Aly, Jifang Wen

**Affiliations:** 1 Pathology and Forensic Sciences department, School of Basic Medical Sciences, Central South University, Changsha, Hunan, China; 2 Forensic Medicine and Clinical Toxicology department, Faculty of Medicine, Suez Canal University, Ismailia, Egypt

Dipterans are attracted to and colonize decomposing corpses immediately after death. From the forensic perspective, their identification is mandatory for postmortem interval estimation. Morphological identification may be difficult or even impossible. Thus, molecular identification has been suggested as an alternative. Many workers suggested that the cytochrome oxidase I (COI) gene is the most suitable gene, but the assignment of a genetic marker within that gene has not been determined yet (1). Previous study has shown the efficacy of a 296-bp sequence in the COI gene in discrimination of species belonging to one dipterous family (Sarcophagidae) from the Czech Republic (2). Because of the genetic variations in insects from different geographical origin, these data should not be applied to any other location until they have been validated (3). Moreover, it is important to examine more than one dipterous family to simulate the condition after database construction (4). Therefore, the present study evaluated the 296-bp COI sequence as mitochondrial genetic marker for identification of dipterous samples from China and Egypt.

We undertook analysis of the most important Chinese taxa associated with cadavers, representing 13 species belonging to Calliphoridae [*Chrysomya megacephala, C. rufifacies, C. albiceps, Lucilia sericata, L. cuprina, L. bazini, L. porphyrina, L. caesar*], Sarcophagidae [*Sarcophaga albiceps*, *S. dux*, *Helicophagella melanura*], and Muscidae [*Musca domestica and M. autumnalis*]. Furthermore, we included the Egyptian species of *L. sericata*, *C. albiceps*, and *M. domestica*, which are considered the most common taxa associated with corpses in Egypt (5). Collection of samples from two geographical locations is very important to evaluate the dual role of these markers not only at the species level but also at the population level. Mitochondrial DNA (mtDNA) from all samples was extracted. The 296-bp fragment of the COI gene from all specimens was amplified using the primers 5′-CAGCTACTTTATGATCTTTAGG-3′ and 5′-CATTTCAAGCTGTGTAAGCATC-3′. Details of the primers and PCR conditions were described by Zehner et al. (2). Then, column cycle sequencing was performed on both forward and reverse strands. A phylogenetic tree based on the COI sequence was constructed by the neighbor-joining (NJ) method using the K2P (Kimura two-parameter) model implemented in the MEGA5 (Molecular Evolutionary Genetics Analysis) and the tree was tested by 1,000 bootstrap replicates.

All tested species displayed 0–1% intraspecific variations. This study showed low intraspecific variation in the three species collected from two different countries, which is in agreement with other previous studies that elucidated the value of mitochondrial genetic markers in general in interspecific distinction (6, 7). The minimum interspecific variations were 4% between *L. sericata*/*L. cuprina* and *C. megacephala*/*C. albiceps*. The maximum interspecific nucleotide divergence was observed between three Sarcophagidae species (10%). The present study showed there was no overlap between maximum intraspecific and minimum interspecific nucleotide divergence. The absence of that overlap was previously interpreted as an indicator of successful species-level resolution (8). At species level, the constructed NJ tree exhibited high bootstrap values that provide robust support for the monophyly of all tested species. Unexpectedly, the two tested Calliphoridae subfamilies (Chrysomyinae and Luciliinae) failed to join together. Chrysomyinae cluster joined with the Muscidae group and Luciliinae cluster joined with the Sarcophagidae group. Thus, these results were not consistent with the taxonomic key-based identification.

In conclusion, although the 296-bp COI marker is a simple, cheap, and reproducible tool, the phylogenetic data were not completely in accordance with the traditional morphological classification, indicating limitation in its usefulness, especially for database construction. This indicates that reliance on this short genetic marker for identification is unsafe.

*Sanaa Mohamed Aly*
Pathology and Forensic Sciences department School of Basic Medical Sciences, Central South University Changsha, Hunan, China Forensic Medicine and Clinical Toxicology department Faculty of Medicine, Suez Canal University Ismailia, Egypt*Jifang Wen* Pathology and Forensic Sciences department School of Basic Medical Sciences Central South University Changsha, Hunan, China
